# Synthetic fibrous hydrogels as a platform to decipher cell–matrix mechanical interactions

**DOI:** 10.1073/pnas.2216934120

**Published:** 2023-04-03

**Authors:** Hongbo Yuan, Kaizheng Liu, Mar Cóndor, Jorge Barrasa-Fano, Boris Louis, Johannes Vandaele, Paula de Almeida, Quinten Coucke, Wen Chen, Egbert Oosterwijk, Chengfen Xing, Hans Van Oosterwyck, Paul H. J. Kouwer, Susana Rocha

**Affiliations:** ^a^Key Laboratory of Molecular Biophysics of Hebei Province, Institute of Biophysics, School of Health Sciences and Biomedical Engineering, Hebei University of Technology, Tianjin 300401, China; ^b^Molecular Imaging and Photonics, Chemistry Department, KU Leuven, Leuven 3000, Belgium; ^c^Institute for Molecules and Materials, Radboud University, Nijmegen 6525 AJ, The Netherlands; ^d^Research Center for Human Tissue and Organs Degeneration, Institute of Biomedicine and Biotechnology, Shenzhen Institute of Advanced Technology, Chinese Academy of Sciences, Shenzhen 518055, China; ^e^Department of Mechanical Engineering, Biomechanics section, KU Leuven, Leuven 3000, Belgium; ^f^Division of Chemical Physics and NanoLund, Department of Chemistry, Lund University, 221 00 Lund, Sweden; ^g^Department of Urology, Radboud Institute for Molecular Life Sciences, Radboud University Medical Center, Nijmegen 6500 HB, The Netherlands; ^h^Prometheus Division of Skeletal Tissue Engineering, KU Leuven, Leuven 3000, Belgium

**Keywords:** biomaterials, mechanobiology, cell–matrix interactions, cellular traction forces, nonlinear mechanics

## Abstract

Cells continuously sense external forces from their microenvironment, the extracellular matrix (ECM). In turn, they generate contractile forces, which stiffen and remodel this matrix. Although this bidirectional mechanical exchange is crucial for many cell functions, it remains poorly understood. Key challenges are that the majority of available matrices for such studies, either natural or synthetic, are difficult to control or lack biological relevance. Here, we use a synthetic, yet highly biomimetic hydrogel based on polyisocyanide (PIC) polymers to investigate the effects of the fibrous architecture and the nonlinear mechanics on cell–matrix interactions. Live-cell rheology was combined with advanced microscopy-based approaches to understand the mechanisms behind cell-induced matrix stiffening and plastic remodeling. We demonstrate how cell-mediated fiber remodeling and the propagation of fiber displacements are modulated by adjusting the biological and mechanical properties of this material. Moreover, we validate the biological relevance of our results by demonstrating that cellular tractions in PIC gels develop analogously to those in the natural ECM. This study highlights the potential of PIC gels to disentangle complex bidirectional cell–matrix interactions and to improve the design of materials for mechanobiology studies.

## Significance

The interactions between cells and their direct environment are crucial for cell fate. Despite recent advances, the highly complex web of biochemical and mechanical contributions is poorly understood. In fact, real progress is hampered by the lack of appropriately controllable matrices and quantitative analysis techniques that cover a range of time and length scales. Here, we use a highly biomimetic synthetic fibrous hydrogel to study and tailor the bidirectional cell–matrix interactions. Using advanced microscopy-based approaches, we acquire a comprehensive picture of how cellular tractions, fiber remodeling, matrix properties, and cellular behavior interact. Complete mapping of cell–matrix interactions at the cellular length scale provides indispensable information for a rational design of better biomimetic materials.

Cells are intimately linked to their surrounding extracellular matrix (ECM); they receive chemical cues and constantly probe and respond to external mechanical properties. Simultaneously, cells can actively modify the mechanical properties of their environment by secreting or remodeling ECM components, which gives rise to a complex mechanical reciprocity that plays a key role in cell migration and development, tissue regeneration and homeostasis, and pathologic changes (1, 2). Cellular actomyosin-related contraction generates forces that propagate over distances many times the cell diameter, allowing cell–cell mechanical communication and rapid transmission of signals at a long distance (3–5). Additionally, cellular contraction leads to large stiffness gradients in the three-dimensional (3D) matrix, i.e., the ECM closer to the cells becomes substantially stiffer due to matrix densification and a stress-induced stiffening response of the matrix (6–13). As the latter is commonly only found in biological matrices, cell-induced matrix stiffening and displacement propagation over large distances have only been observed in collagen and fibrin matrices (14, 15). Although biocompatible, these matrices are relatively difficult to tailor (16), particularly without modifying the protein concentration, which inherently changes other matrix characteristics too (e.g., pore size and concentration of adhesion ligands). Moreover, such biological matrices are sensitive to enzymatic degradation, which will change matrix properties in time.

Synthetic hydrogels are uniquely suited to decouple biochemical and mechanical cell–matrix interactions. In this work, we recreated the mechanical cell–matrix interactions observed in biological materials in a fully synthetic hydrogel based on polyisocyanides (PICs), which allowed us to study the cell–matrix reciprocity in a very selective and controlled fashion. Using a combination of 3D displacement microscopy, equivalent to traction force microscopy (TFM), and live-cell rheology, we delineated characteristic time and length scales of displacement propagation and matrix stiffening, which is key information for the understanding of cell–matrix mechanical interactions and rational design of the next generation of biomimetic materials.

## Results

### Cell-Mediated Mechanical Remodeling.

**Fig. 1.**
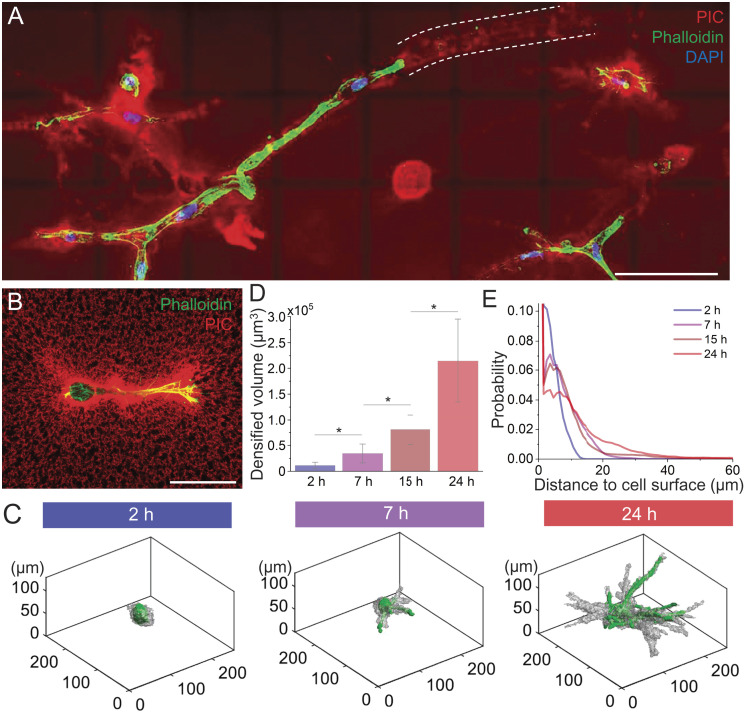
Cell-mediated mechanical remodeling in PIC matrices. (*A*) Maximum projection image of hASCs in a fluorescently labeled ${\mathrm{PIC}}_{Short}^{RGD+}$ gel 7 d after encapsulation (different PIC gels are discussed in the next section). White dotted lines indicate the tunnels formed by cell migration that remain in the PIC matrix. (Scale bar, 100 µm.) (*B*) Higher-resolution fluorescence image of a hASC in the ${\mathrm{PIC}}_{Short}^{RGD+}$ network 24 h after incubation. (Scale bar, 50 µm.) Red: PIC fibers. Green: phalloidin. Blue: DAPI. (*C*) 3D render images of representative cells, 2, 7, and 24 h after encapsulation, displaying the cell surface (green) and the volume of remodeled matrix (gray). The remodeled matrix was determined using an intensity-based threshold (details in the *Materials and Methods* section). (*D*) Evolution of the densified fiber volume through time (N = 5, *P < 0.05, by one-way ANOVA Tukey’s test). (*E*) Distribution of the fiber remodeling range with incubation time. The PIC matrix used in these experiments was ${\mathrm{PIC}}_{Short}^{RGD+}$ with short polymer lengths at 1.0 mg mL^−1^, yielding gels with a low critical stress and high sensitivity to cellular forces (parameters in Table 1).

The PIC-based hydrogels used here combine the structural and mechanical characteristics of a biological gel, with the tailorability of a synthetic gel (17–20). Decoration with the generic cell-adhesive peptide GRGDS (PIC*^RGD^*) makes the material particularly suitable as a matrix for the 3D culture of different cells and organoids (21–23), where control over (mechanical) matrix properties is used to direct cell fate, such as stem cell differentiation (24), cell morphogenesis (21, 25), and the secretome (26). Live-cell imaging of human adipose-derived mesenchymal stem cells (hASCs) in fluorescently labeled PIC*^RGD^*^+^ (see the *Materials and Methods* section and *SI Appendix*, Fig. S1 for details) shows cell migration and matrix remodeling, as indicated by the increased fluorescence intensity of the matrix close to the cell (Fig. 1 *A* and *B* and Movie S1). In addition, it is possible to observe channels formed by migrating cells (Fig. 1*A*, dotted lines). Since the PIC network is not chemically cross-linked but held together with noncovalent interactions (17), we hypothesize that the observed remodeling is purely mechanical and results from mechanical cell–matrix interactions, in line with the migration mechanism described for alginate-based gels (9). Plasticity of the matrix ensures that the formed channels remain in the material. Further zooming in on the early (24 h after incubation) remodeling process taking place around the cell, the images acquired show a clear fiber densification and reorganization throughout the matrix (Fig. 1*B*).

To quantitatively investigate the dynamics and extent of cell-induced fiber remodeling with time, regions displaying fiber densification were defined using an intensity-based threshold, following the approach described by Lesman and coworkers (14) (Fig. 1 *C* and *D*). Two hours after cell encapsulation, the extent of remodeling is almost negligible, but after that, the matrix volume that shows cell-induced fiber densification gradually increases and the distance of remodeled matrix, as measured from the cell surface, increases from less than 10 µm at 2 h to more than 50 µm after 24 h (Fig. 1*E* and *SI Appendix*, Fig. S2). Despite being a common phenomenon in natural matrices, such long-range mechanical remodeling of matrix fibers is rarely reported in a fully synthetic material.

### Cell-Induced Fiber Displacements and Propagation.

To evaluate cellular force–induced matrix displacements and remodeling, we monitor changes in the fiber architecture of the PIC gel after the removal of the contractile forces by addition of the actin-disrupting agent cytochalasin D (cytoD) (27, 28). In contrast to conventional TFM, there is no need to add particles to indirectly monitor matrix displacements: The fluorescently labeled PIC gels allow direct bead-free tracking (*SI Appendix*, Fig. S1).

**Fig. 2.**
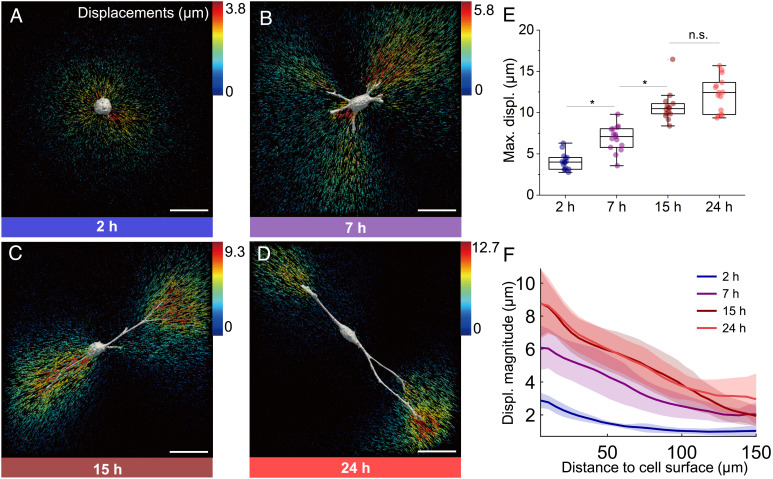
Cellular force–generated fiber displacements and transmission range in PIC matrices. (*A*–*D*) 3D displacement field induced by hASCs encapsulated in ${\mathrm{PIC}}_{Short}^{RGD+}$ gel after the addition of cytoD at different time points (2, 7, 15, and 24 h, respectively). The arrows indicate the calculated displacement of the PIC fiber network and are color-coded according to the magnitude of the local displacement vector (in µm). Notice the different color scales for *A*–*D*. (Scale bars, 50 µm.) (*E*) The maximum displacement (max. displ.) plotted for each cell at different incubation times (N = 12 to 15, *P < 0.05, by one-way ANOVA Tukey’s test). The box plots depict minimum, first quartile, median, third quartile, and maximum values. (*F*) Plots of the amplitude of the fiber displacements as a function of distance to the cell surface at different time points. The SD is represented as a shaded area for each line. ${\mathrm{PIC}}_{Short}^{RGD+}$ with a default concentration 1.0 mg mL^−1^ was used (Table 1).

Three-dimensional cultures of hASCs containing a fluorescent dye (CellTracker™ Green) encapsulated in the GRGDS-decorated and fluorescently labeled PIC hydrogel were imaged after 2, 7, 15, or 24 h of incubation. The cells were then relaxed by adding cytoD and imaged again (*SI Appendix*, Fig. S3 and Movie S2). Fiber displacements between the stressed and relaxed states of the cell were measured using the open-source 3D TFM toolbox TFMLAB to generate matrix displacement maps (29). The arrows in the displacement maps indicate the direction and magnitude of the measured displacements (Fig. 2 *A*–*D*, details in the *Materials and Methods* section).

The measurement of cell-induced matrix displacements with time provides quantitative information on the dynamics of cell–matrix interactions (30). Two hours after encapsulation, the cells are mostly spherical and apply uniform, radially oriented traction forces on its surroundings, pulling the fiber network toward the center of the cell (Fig. 2*A*), which is in line with the observed patterns of fiber remodeling. With time, cells spread and develop protrusions (Fig. 2 *B*–*D*), which gradually become longer and slenderer. We find that the largest matrix deformations are present at the tips of these protrusions. To quantify the evolution of matrix displacements through time, the maximum fiber displacement for each cell was determined (Fig. 2*E*). During the first 15 h in culture, the average maximum displacement gradually increases over time until reaching a plateau where cells are considered stable in the gel as no significant differences in displacements can be detected after 15 h of incubation.

The range of cell-induced displacements into the fibrous matrix was calculated by plotting fiber displacements as a function of the distance from the cell surface (Fig. 2*F*). After 7 h in culture, we detect fiber displacements as far as 150 µm from the cell surface^*^, which is in strong contrast to values that have been reported using linear elastic materials (such as polyethylene glycol or polyacrylamide gels) where the displacement amplitudes rapidly decay within a distance at the same order of the cell diameter (approximately 20 µm) (31, 32).

In addition, the formation of bundles of aligned fibers between cells in close proximity was also observed (*SI Appendix*, Fig. S4), a phenomenon that was reported only in biological matrices like reconstituted collagen (33) and fibrin (14) gels. Such aligned fiber structures are known to be involved in long-range mechanocommunication and molecular transport between cells in 3D environments (4) and required the unique, nonlinear responses of fibrous networks present in the natural ECM (3).

### Cell-Mediated Matrix Stiffening and Plastic Remodeling.

Cell-induced matrix stiffening, as earlier observed in natural biopolymer networks such as collagen, fibrin, and Matrigel (6, 7), is usually attributed to the combination of contractile cellular forces and the nonlinear elastic behavior of the materials. The contribution of matrix remodeling, including fiber densification, is often overlooked. Here, we combine live-cell rheological tests and fluorescence image analysis to unravel the role of cellular forces and fiber remodeling in cell-induced matrix stiffening.

**Fig. 3.**
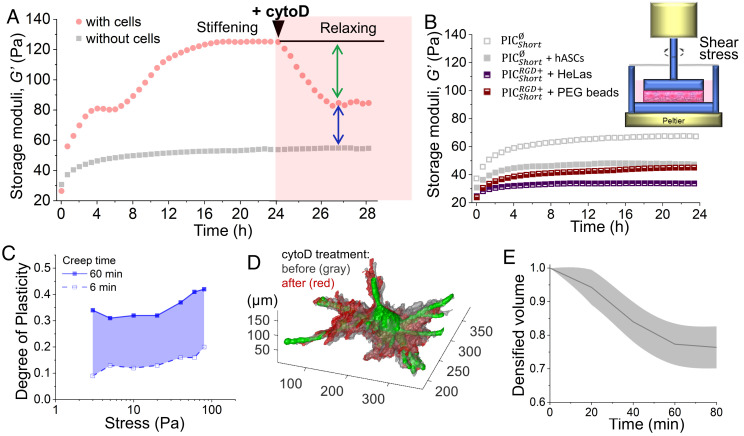
Cell-mediated matrix stiffening and plastic remodeling in PIC matrices. (*A*) The storage modulus *G*′ of ${\mathrm{PIC}}_{Short}^{RGD+}$ gels with (red dots) or without hASCs (gray squares) were monitored in real time through live-cell rheological measurement. The *Left* panel indicates the cell-induced stiffening behavior for the first 24 h before adding cytoD, and the *Right* panel in pink indicates the relaxing effect for an additional 4 h after adding cytoD (5 µM). The green arrow indicates the decrease in *G*′ caused by the addition of cytoD, while the blue arrow indicates the irreversible change in *G*′ (compared to the sample without cells). (*B*) Mechanical properties of gels that do not stiffen: PIC without RGD ( ${\mathrm{PIC}}_{Short}^{\emptyset }$ ) with and without hASCs and ${\mathrm{PIC}}_{Short}^{RGD+}$ with less-contractile HeLa cells and with PEG beads. Inset: Illustration of the setup used for live-cell rheology. A cold solution of PIC with or without cells was placed inside a shear rheology setup with a petri dish–like plate geometry. The solution was warmed to 37 °C to form a gel, and then warm CO_2_-independent medium was added into the dish. HeLas and hASCs in RGD-free gels maintain a spherical morphology (*SI Appendix*, Fig. S6). (*C*) Degree of plasticity of ${\mathrm{PIC}}_{Short}^{\emptyset }$ gels as a function of stress with different creep times. Solid line: 60 min and dashed line: 6 min. At increasing timescales, PIC gels exhibit higher degrees of plasticity. (*D*) Representative 3D rendering of the remodeled matrix before (gray) and 1 h after (red) adding cytoD. The initial image was acquired 24 h after incubation. The cell surface is depicted in green. (*E*) Evolution of the densified fiber volume over time after adding cytoD (N = 5). The SD is represented as a shaded area. Both ${\mathrm{PIC}}_{Short}^{RGD+}$ and ${\mathrm{PIC}}_{Short}^{\emptyset }$ polymers were used at a concentration of 1.0 mg mL^−1^.

The storage modulus *G*′ of PIC gels containing hASCs increases until it plateaus after approximately 15 h (Fig. 3*A*, red dots). The final storage modulus at 24 h is about three times higher than *G*′ of the gel without cells (gray squares) and remains relatively constant up to 48 h (*SI Appendix*, Fig. S5*A*). Lower cell densities reduce the stiffening effect (*SI Appendix*, Fig. S5*B*). For stiffening, both contractile cells, like hASCs, and good cell–gel interactions (through RGD) are required. For instance, PEG microspheres (20 µm diameter) and less-contractile HeLa cells do not induce stiffening (Fig. 3*B*). Also, hASCs seeded in a PIC gel without RGD ( ${\mathrm{PIC}}_{Short}^{\emptyset }$ in Table 1) are unable to induce stiffening. In fact, micron-sized objects that do not induce stiffening actually give rise to a small decrease in *G*′ as a result of reduced entanglements in the matrix (34). The timescale of matrix deformation observed using fluorescence microscopy (Fig. 2*E*) coincides with the macroscopic stiffening response measured by rheology, suggesting that both effects are caused by cellular tractions.

**Table 1.** Characterization of the library of PIC gels, collagen, and matrigel matrices used

Matrix	*L*_c_ (nm)^*^	*c* (mg mL^–1^)	GRGDS density (µm)	Shear modulus *G*′ (Pa)^†^	Critical stress *σ*_c_ (Pa)^†^
${\mathrm{PIC}}_{Short}^{\emptyset }$	154	1.0	–	47	10.6
${\mathrm{PIC}}_{Short}^{RGD}$	154	1.0	3.7	46	8.6
${\mathrm{PIC}}_{Short}^{RGD+}$	154	1.0	31.4	45	7.7
${\mathrm{PIC}}_{Long}^{RGD+}$	229	1.0	31.4	93	19.9
1.5 × ${\mathrm{PIC}}_{Short}^{RGD+}$	154	1.5	47.1	89	11.6
Collagen	n/a^‡^	1.2	n.d.^§^	60	3.5
Matrigel	n/a^‡^	8 to 11^¶^	n.d.^§^	28	6.9

^*^Polymer contour length, *L*_C_, was calculated from the viscosity-based molecular weight.

^†^Mechanical properties were measured in α-MEM at 37 °C in absence of cells.

^‡^Not applicable.

^§^Not determined.

^¶^Typical protein concentrations for Corning Matrigel matrix are between 8 and 11 mg mL^–1^.

Cell contraction, and the resulting macroscopic stiffening response, was terminated by the addition of cytoD, which decreased *G*′ but not to the value without cells (Fig. 3*A*, red dots). We hypothesize that the remaining stiffening is caused by plasticity-mediated matrix remodeling, i.e., the architectural reorganization of the polymer network induced by cellular remodeling. Due to the plasticity of the PIC network, full recovery of the remodeled structure after cell relaxation is prevented. Plasticity is qualitatively inferred from the cell migration channels in the fluorescence images (Fig. 1*A*). We then studied if plastic remodeling of the matrix directly affects the mechanical properties. We performed a series of macroscopic creep and recovery tests (*SI Appendix*, Fig. S7) and found that while the degree of plasticity, defined as the portion of irreversible change in strain after the creep and recovery test, increases with applied stress (Fig. 3*C*), the stiffness of the matrix remains constant for all plastic deformations.

To quantify the plastic character of the cell-induced matrix remodeling at the micrometer scale, we traced and analyzed time-lapse fluorescence images of the fiber network after inhibiting cellular contraction. CytoD addition reduced the length of the cellular protrusions as the cell reached the stress-free state (Fig. 3 *D* and *E*). Simultaneously, the volume of densified PIC matrix gradually decreased until approximately 25% of the initial densified volume, which indicates that a large fraction of the matrix surrounding the cell is deformed plastically and irreversibly (∼75%, see Fig. 3*E*). We attribute the residual macroscopically observed stiffening after cytoD addition to the (local) permanently remodeled architecture of the PIC matrix. Overall, we conclude that the observed matrix stiffening has two origins, both induced by cellular contraction: a fast nonlinear strain-stiffening response that is fully reversible and a slower and irreversible plastic remodeling response that includes fiber densification and reorganization.

### Nonlinear Mechanics of the Fibrous Network Dominates the Cell–Matrix Mechanical Interactions.

The nonlinear mechanics of PIC gels are readily tuned by the polymer concentration, *c*, and also at constant *c* by the polymer contour length (*L*_c_) (19). For instance, for gels of shorter polymers, the critical stress *σ*_c_ (the onset of nonlinear stiffening) decreases, which gives rise to a material that is more sensitive to stress (19). Fluorescence microscopy on labeled PIC gels, however, showed that the fiber architecture at the micrometer scale depends on *c*, but is not affected by *L*_c_ (35), which allows one to tune the mechanical properties without affecting the gel microstructure.

Here, we exploited the tailorability of the PIC gel to learn more about cell–matrix interactions. hASCs were encapsulated (3D) in three different PIC gels. Two types of gel comprised short PIC polymers, with medium and high GRGDS densities ( ${\mathrm{PIC}}_{Short}^{RGD}$ and ${\mathrm{PIC}}_{Short}^{RGD+}$ ), respectively, and a third gel, made of long PIC polymers, with a high GRGDS density ( ${\mathrm{PIC}}_{Long}^{RGD+}$ ). Gels prepared from short polymer exhibit a low critical stress, which indicates a higher sensitivity to mechanical or cell-induced stress. In contrast, long PIC polymers form gels with a higher critical stress, presenting lower mechanical sensitivity. The properties of the gels used are summarized in Table 1.

**Fig. 4.**
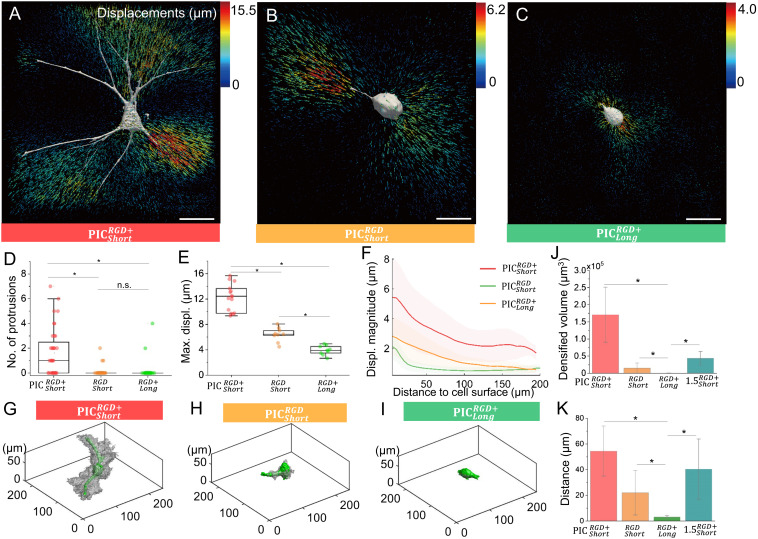
Influence of the biological/mechanical properties on cell–matrix mechanical interactions. hASC-induced 3D matrix displacements in ${\mathrm{PIC}}_{Short}^{RGD+}$ (*A*), ${\mathrm{PIC}}_{Short}^{RGD}$ (*B*), and ${\mathrm{PIC}}_{Long}^{RGD+}$ (*C*) gels at 24 h after incubation. Arrows indicate displacement directions and are color-coded for magnitudes (notice the different color scales for *A*–*C*). (Scale bars, 50 µm.) (*D*) Number of protrusions in hASCs in different PIC gels (N = 20 to 25). (*E*) Maximum cell-induced matrix displacement (max. displ.) in the different PIC gels (N = 12 to 15). The box plots in panels d and e depict minimum, first quartile, median, third quartile, and maximum values. (*F*) Plots of fiber displacements as a function of distance from the cell surface (N = 8 to 10). The SD is represented as a shaded area for each line. (*G*–*I*) Plots of a representative cell in different PIC gels, displaying the cell body (green) and the volume of remodeled matrix (gray). The densified volumes were detected using an intensity-based adaptive threshold (details in the *Materials and Methods* section). (*J*) Densified fiber volume in different PIC matrices (N = 5). (*K*) Maximum distance of the detected edge of fiber densification for the different PIC matrices (N = 5). 1.5 × ${\mathrm{PIC}}_{Short}^{RGD+}$ refers to the sample prepared with short ${\mathrm{PIC}}_{Short}^{RGD+}$ polymers but with a higher concentration (1.5 mg mL^−1^) compared to the default condition in other experiments (1.0 mg mL^−1^), see the detailed parameters in Table 1. *P < 0.05, by one-way ANOVA Tukey’s test.

Twenty-four hours after encapsulation, live-cell imaging was performed to evaluate cell morphology, fiber displacements, and the degree of matrix remodeling (Fig. 4). In line with previous work (21), we found that the spreading of hASCs is stronger in RGD-rich soft matrices ( ${\mathrm{PIC}}_{Short}^{RGD+}$ ) characterized by the long and numerous slender protrusions and an elongated morphology (Fig. 4*A* and *SI Appendix*, Fig. S8). Spreading reduces with decreased concentration of adhesion ligands or in a stiffer matrix (Fig. 4 *B* and *C*). Image analysis of 12 to 15 representative cells confirms that cells in ${\mathrm{PIC}}_{Short}^{RGD+}$ form more protrusions (Fig. 4*D*) and increased levels of fiber displacement and propagation distance (Fig. 4 *E* and *F*).

Matrix stiffening as a result of plastic remodeling is directly related to the volume of the remodeled matrix around the cells. Further image analysis allows us to generate a full 3D remodeling field around the hASCs in each matrix (Fig. 4 *G*–*I*, the averaged data of densified volume and distance to the cell surface are shown in panels *J* and *K*). The matrix displays more pronounced remodeling in ${\mathrm{PIC}}_{Short}^{RGD+}$ and much less for the gels containing less RGD or stiffer gels with the same polymer concentration but longer chains. In addition, we note that while the amplitude of the matrix deformation in ${\mathrm{PIC}}_{Short}^{RGD}$ was lower compared to ${\mathrm{PIC}}_{Short}^{RGD+}$ , the range of propagation and decay of the cellular force–induced displacements was similar in both gels (Fig. 4 *E* and *F*). In contrast, in the ${\mathrm{PIC}}_{Long}^{RGD+}$ gels, fiber displacements were only detected in the pericellular region, rapidly decreasing within 30 µm from the cell surface, which suggests that the force transmission range mainly depends on the mechanical properties of the matrix and is less affected by the number of cell–matrix adhesion sites.

The differences observed between hydrogels composed of long and short polymer chains can be linked to higher critical stress or higher storage modulus (Table 1). While it is possible in PIC gels to selectively tune the critical stress while keeping the storage modulus constant (19), this procedure requires a variation in concentration and therefore affects the microstructure of the gel (35). To zoom in on the contribution of the nonlinear mechanics, we prepared a gel of ${\mathrm{PIC}}_{Short}^{RGD+}$ at a slightly higher polymer concentration (*c* = 1.5 mg mL^−1^, labeled as 1.5×${\mathrm{PIC}}_{Short}^{RGD+}$ in Table 1), with a shear modulus *G*′ comparable to ${\mathrm{PIC}}_{Long}^{RGD+}$ (at *c* = 1.0 mg mL^−1^) but a lower critical stress (11.6 versus 19.9 Pa, see Table 1). Although a higher polymer concentration will lead to smaller micron-sized pores, cells can still well spread compared to the long-polymer PIC samples. Fluorescence microscopy after 24-h incubation (*SI Appendix*, Fig. S9) shows that hASCs embedded in 1.5 × ${\mathrm{PIC}}_{Short}^{RGD+}$ aligned and densified the matrix. While the densified volume was smaller than that in ${\mathrm{PIC}}_{Short}^{RGD+}$ , it was still significantly larger than that in the ${\mathrm{PIC}}_{Long}^{RGD+}$ gels (Fig. 4 *J* and *K*), which suggests that the nonlinear mechanics of PIC gels plays an important role in the cell–matrix interactions. We propose that for the long-chain PIC gels, the threshold stress at which the polymers slide and merge to remodel is beyond the range of hASCs-generated forces, and therefore, the amount of fiber remodeling is greatly reduced for increasing values of critical stress.

### Comparison with Biological Matrices.

To analyze the biological relevance of our analysis on synthetic matrices, we benchmark our results to cell-induced matrix displacements in natural matrices. hASCs were encapsulated in collagen and Matrigel matrices; we aimed to roughly match the linear and nonlinear mechanical properties of the biopolymer networks to ${\mathrm{PIC}}_{Short}^{RGD+}$ , despite the strong differences in strain-stiffening effects in the three matrices (Table 1).

**Fig. 5.**
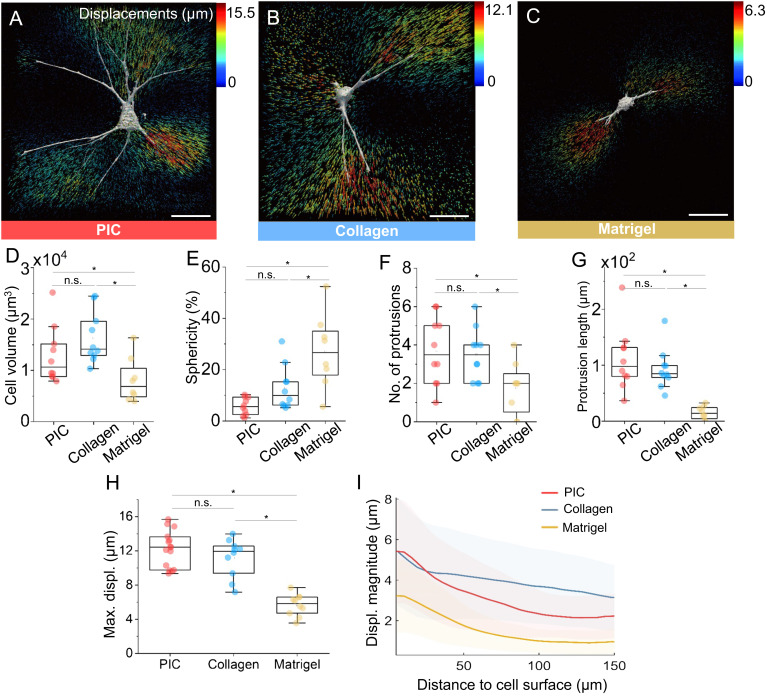
Comparison of cell behavior and cell–matrix mechanical interactions between PIC and biological matrices. hASC-induced 3D matrix displacement fields in PIC ( ${\mathrm{PIC}}_{Short}^{RGD+}$ , 1.0 mg mL^−1^) gel (*A*), collagen type I (1.2 mg mL^−1^, *B*), and Matrigel (70%, *C*). The color of the arrows indicates the magnitude of the local displacement vector. (Scale bars, 50 µm.) Comparison of cell morphology in different matrices after 24-h incubation, including cell volume (*D*) and sphericity (*E*), protrusion number (*F*), and length (*G*). (*H*) Maximum cell-induced matrix displacement (max. displ.) in different matrices. For all box plots, each point refers to the data of one cell and depict minimum, first quartile, median, third quartile, and maximum values. (*I*) Plots of matrix displacements as a function of distance to the cell surface. The shaded areas along the curves indicate SD. For each group, N = 8 to 12, *P < 0.05, by one-way ANOVA Tukey’s test.

Twenty-four hours after encapsulation, hASCs displayed a well-spread morphology in both PIC and collagen matrices while spreading was less in Matrigel (Fig. 5 *A*–*C*). Cells in PIC and collagen show a similar cellular sphericity, volume, number of protrusions, and protrusion length, while in Matrigel, the cells are less spread, showing smaller protrusions (Fig. 5 *D*–*G*). Displacement analysis reveals that the fiber displacement field observed in PIC hydrogels is close to that observed in collagen (Fig. 5 *A* and *B*) with no significant differences on the calculated maximum displacement (Fig. 5*H*). Remarkably, the displacement propagation was also similar in both matrices (Fig. 5*I*); both PIC and collagen gels support a long-range fiber displacement propagation, reaching over hundred microns from the cell surface. However, the decay rate of the displacement varies considerably between the two gels. According to previous theoretical studies, this difference can be attributed to variations in fiber stiffness or elastic anisotropy (36, 37). In fact, while both gels possess a fibrous structure, collagen gels exhibit much thicker fiber dimensions (200 to 400 nm) compared to PIC gels (10 to 20 nm). In contrast, Matrigel is a nonfibrous matrix. The fewer and shorter protrusions in Matrigel generated a much smaller and more local displacements (note the different color scales in Fig. 5 *A*–*C*).

In general, cell spreading is affected by matrix mechanics, network porosity, and integrin-mediated cell–matrix interactions (38). Similar to collagen, PIC gels present a fibrous architecture with pore size in the micrometer range (35). The α_5_β_1_ integrin dimer, highly abundant in hASCs, binds well to RGD and to collagen type I (39, 40), which gives rise to pronounced cellular spreading and an extensive matrix remodeling. Matrigel, on the other hand, exhibits a much denser and sterically constrained matrix (41), with laminin as the main component, which interacts primarily with α_3_β_1_ integrins (that are less expressed in hASCs) (39, 40). We observe that the displacement propagation through the Matrigel matrix resembles the decay observed in RGD-reduced PIC gels ( ${\mathrm{PIC}}_{Short}^{RGD}$, Figs. 4*E* and 5*I*), which seems to suggest that the reduced cell spreading and smaller fiber displacements observed in Matrigel is a consequence of lesser cell–matrix interactions.

## Discussion

Over the last two decades, a plethora of studies have demonstrated unambiguously that the mechanical properties of the cell’s environment determine its fate. While it is often easy to measure these properties macroscopically, it remains very difficult to analyze them in 3D samples at the micron scale, i.e., at the cellular length scale, particularly in fibrous matrices such as collagen, which are not homogeneous at this scale. In this manuscript, we showed that cells bind to a matrix and apply contractile forces to it, actively modifying their environment, even when it is nondegradable. With a combination of macroscopic (live cell) mechanical analysis and microscopic 3D displacement measurements, we were able to map how cellular contractile forces lead to remodeling and stiffening through two distinct mechanisms. At short timescales, cellular contraction deforms the matrix, which instantaneously results in a stiffening response due to the nonlinear strain-stiffening properties of the matrix, which is directly linked to its fibrous architecture. This effect is temporary, i.e., as the stress is removed, for instance, by cytoD addition or at longer timescales by viscoelastic dissipation (16), matrix stiffening disappears.

At longer timescales, cells are able to remodel their matrix, densifying and contracting it. In fibrous matrices, the results of remodeling are not only noticed close to the cell, but the effect can reach out hundreds of microns into the matrix, which sets up a mechanism for easy long-range cellular communication. The high plasticity of the matrix makes remodeling (partially) permanent, and even after removal of cellular tractions, the local matrix densification and reorganization remain, leading to matrix changes that can be macroscopically traced.

To study biologically relevant cell–matrix interactions, one ideally incorporates as many as possible characteristics of the in vivo microenvironment. From our work, it becomes increasingly clear that key design features include a fibrous architecture (preferably with associated nonlinear mechanical properties) and sufficient cell–matrix interactions to achieve a reliable traction response. Most likely, the latter requires optimization for each cell type (42). In that respect, synthetic materials are particularly attractive as matrices because of their high customizability, both mechanically and biochemically, which can also be used to drive desired effects. When studying at longer timescales, also effects of matrix degradation and the deposition may be considered.

Taken together, the interdisciplinary combination of a customizable biomimetic material and advanced fluorescent microscopy techniques has the potential to become a highly valuable cell biology toolbox to study the reciprocal cell–matrix interaction in dilute systems as well as more complex heterocellular systems.

## Materials and Methods

### Polymer Synthesis and Functionalization.

#### PIC polymer synthesis.

PICs were synthesized using an established protocol as reported previously (21). The polymer contour length, *L*_C_, was controlled by adjusting the total monomer-to-catalyst ratio and estimated from the viscosity-average molecular weight. All azide-modified polymers carried 3.3% azide monomers and 96.7% methoxy-terminated monomers.

#### Peptide conjugation.

A linear GRGDS peptide with BCN [bicyclo(6.1.0)nonyne] was used. The linker between the peptide and the BCN reactive handle was PPPSG[Abz]SG, where Abz is 4-aminobenzoic acid. Peptides were purchased from Pepsan, the Netherlands. The azide-decorated polymer was first dissolved in acetonitrile (2.5 mg/mL), and the appropriate volume of the peptide solution was added (6.0 mg/mL in dimethyl sulfoxide). The solution was stirred for 24 h at room temperature. Next, the polymer–peptide conjugates were precipitated in diisopropyl ether, collected by centrifugation, and air-dried for 2 to 3 d.

#### Fluorescence labeling.

To fluorescently label the polymers, a red-emitting fluorescence dye Atto647N with covalently grafted. Atto647N-NHS ester was conjugated with DBCO-PEG4-amine (Sigma Aldrich), yielding DBCO–Atto647N. This compound was added to the polymer solution in a ratio of 1 mg polymer:0.5 nmol dye, incubated for 5 min on ice, and then used for cell encapsulation.

### Cell Culture.

#### Cell passage.

hASCs were isolated from patients with informed consent, stored in the Radboud University Medical Center Urology Biobank (The Medical Ethics Review Committee of the Oost-Nederland Ethics Committee, approval CWOM9803-0060), and deidentified prior to use in this study. The cells were grown in α-MEM (Gibco, Thermo Fisher, USA) supplemented with 10% fetal bovine serum (FCS) (Gibco, Thermo Fisher, USA) and 1% penicillin/streptomycin (10,000 units/mL penicillin and 10,000 µg/mL streptomycin, Gibco, Thermo Fisher). The cells from the same patient sample and the same passage number (p = 6) were used for experiments. The HeLa cells were cultured in RMPI 1640 media (Gibco, Thermo Fisher) supplemented with 10% FCS (Gibco, Thermo Fisher, USA) and 1% *L*-glutamine (Gibco, Thermo Fisher, USA). Cells were incubated at 37 °C and 5% CO_2_.

#### Encapsulation in PIC gels.

Cells were encapsulated in PIC gels by mixing a cell suspension and polymer solution (v/v = 1:1) at a lower temperature (on ice) and warming up to 37 °C in an incubator. The initial PIC polymer solution was 2.0 mg/mL, resulting in a final concentration of 1.0 mg/mL (except when specified otherwise). The final cell density was 10^6^ cells/mL for live-cell rheology experiments, 8 × 10^4^ cells/mL for the analysis of cell morphology, and 4 × 10^4^ cells/mL for TFM. Imaging was performed using angiogenesis plates (Ibidi). In agreement with previous reports (26), at this concentration, the majority of the cells displayed protrusions within 24 h after encapsulation (Movie S1).

#### Encapsulation in collagen gels.

The collagen gels used in this work were prepared using a protocol previously published (43). Briefly, to prepare 310 µL of 2.4 mg/ml collagen type I hydrogels, we mixed 125 µL of rat tail collagen (Collagen R, 2 mg/mL, Matrix BioScience), 125 µL bovine skin collagen (Collagen G, 4 mg/mL, Matrix BioScience), 28 µL NaHCO_3_ (23 mg/mL), 28 µl 10× α-MEM, and 4 µL NaOH (1 M) to adjust the pH to 10. The final collagen concentration of 1.2 mg/mL was prepared by diluting the initial solution with 310 µL of a mixture of 1 volume part of NaHCO_3_ (23 mg/mL), 1 part of 10× α-MEM, and 8 parts of distilled H_2_O. The pH was adjusted to 10 using NaOH (1 M). Cells were carefully mixed with the final collagen on ice to a final density of 4 × 10^4^ cells/ml and added to angiogenesis plates (Ibidi). The gel was formed by placing the sample in a cell culture incubator at 37 °C, 5% CO_2_, and 95% humidity for 1 h.

#### Encapsulation in matrigel.

A Corning^®^ Matrigel^®^ matrix (reference 356231) was used. 70% Matrigel and 30% of cell solution with desired density were mixed on ice. The gel–cell mixture was then pipetted into the precoated Ibidi µ-slide and incubated for 30 min at 37 ˚C to allow gelation.

### Fluorescence Staining.

At 24 h after encapsulation, gels were washed with phosphate-buffered saline (PBS) and then fixed with 4% paraformaldehyde in PBS for 40 min. After fixation, the samples were permeabilized with 0.1% Triton X-100 in PBS for 10 min and blocked with 1% BSA in PBS for 30 min. The sample was incubated with Phalloidin Atto-520 (1:20 in 1% BSA/PBS, 10 μM, Sigma-Aldrich, USA) for 1 h for staining the actin fibers. After this, the cells were stained with Hoechst (40 µM) for 30 min and washed with PBS (3×). All procedures were performed at 37 °C. The images were acquired using a Leica SPX8 microscope and equipped with a water objective (HC PL APO 63×/1.40, motCORR, Leica) and a HYD-SMD detector. As excitation sources, a supercontinuum white light laser (470 to 670 nm, pulsed, 80 MHz, NKT Photonics) and a UV diode laser (405 nm, pulsed, 40 MHz, PicoQuant) were used. All measurements were performed in a temperature-controlled environment (37 °C).

### Rheology.

#### Mechanical characterization of PIC gels.

Both the linear and nonlinear mechanical properties of PIC gels were characterized as previously reported (19). Briefly, a stress-controlled rheometer (Discovery HR-2, TA Instruments) with an aluminum or steel parallel plate geometry was used (diameter = 40 mm and gap = 500 μm) to measure the mechanical properties of the hydrogels. Polymer solutions were loaded onto the rheometer plate at *T* = 5 °C, followed by a temperature ramp to *T* = 37 °C at a rate of 1.0 °C min^–1^. In the linear viscoelastic regime, the moduli were measured at an amplitude of *γ* = 0.02 and a frequency of *f* = 1.0 Hz. Prior to the nonlinear measurements, the polymer solution was allowed to equilibrate for 30 min at 37 °C. Here, the hydrogels were subjected to a constant prestress of *σ*_0_ = 0.5 to 200 Pa, and the differential modulus *K′* was determined with a small superposed oscillatory stress at frequencies of *f* = 10 to 0.1 Hz (reported data at *f* = 1 Hz). The oscillatory stress was at least 10 times smaller than the applied prestress. In addition, creep and recovery tests were performed to characterize the plasticity of PIC gels. A constant stress ranging from 5 to 80 Pa was applied for 300 to 3,600 s, while strain in response to the stress was measured over time. Then, the stress was removed in recovery tests, and the strain was monitored until it reached equilibrium (*SI Appendix*, Fig. S10). The degree of plasticity is defined as the ratio of irreversible strain after recovery tests to the maximum strain at the end of the creep tests (44).

#### Live-cell rheology.

A custom-made aluminum lower petri dish–like plate geometry (inner diameter 45 mm and height 10 mm) was designed and used to measure the mechanical properties of the cell–gel constructs. A cold cell–polymer solution (0.6 mL) was loaded into the dish center at 5 °C, and the upper plate (20 mm) was lowered to a 1,000-µm gap. Once the temperature was raised to 37 °C, the gel containing the cells was allowed to stabilize for 5 min. Then, warm CO_2_-independent medium (5 mL Gibco, Thermo Fisher, USA) was added into the dish. Next, a solvent trap cover was mounted to create a thermally stable vapor barrier. The storage modulus *G′* was determined by applying an oscillating deformation of amplitude *γ* = 2% with *f* = 1 Hz in a time sweep for 24 to 48 h. For the cell–gel rheology with cytoD experiments, 5 mL of 10 µM cytoD in warm α-MEM was added to the system during the pausing procedure at 24 h, and then, the measurement continued. PEG microspheres were kindly provided by Prof. Laura de Laporte (RWTH Aachen, Germany) with an average diameter of 20 µm. The seeding densities of live cells and PEG microspheres are both set to 10^6^/mL.

### 3D Displacement Microscopy.

For cell traction–induced 3D matrix displacement measurements (3D displacement microscopy), hASCs were incubated for 45 min with 12.5 µM CellTracker™ Green CMFDA (5-chloromethylfluorescein diacetate) (Life Technologies, Belgium) in serum-free α-MEM 1 d before gel encapsulation. PIC polymers were labeled with DBCO-Atto647N to fluorescently label the fiber network. The volume ratio between DBCO-Atto647N:cell solution:polymer solution was 1:9:10. Briefly, the dye (10 μM) and the polymer solution (2 mg/mL) were mixed on the ice first, and then, the cold cell solution (80,000 cells/mL) was added and mixed gently. 12 µL of the mixture was pipetted in a µ-Slide angiogenesis dish (Ibidi) and warmed up in an incubator to create cell–gel constructs. Collagen gel samples were imaged using second harmonic generation (SHG) microscopy (45, 46). For Matrigel samples, the gel deformation was tracked using embedded fluorescence beads (FluoSpheres™, 0.2 µm, red fluorescent (580/605), Thermo Fisher, F8810). A cell density of 4 × 10^4^ cells/mL was used for the different matrices. This low density minimized the influence of neighbor cells on the observed displacements. Furthermore, the images were closely inspected during the segmentation and displacement recovery steps of the algorithm to confirm the absence of any protrusions from other cells or rogue fiber displacements.

#### Image acquisition.

For PIC gel samples, fluorescence images were acquired using a Leica SP8 confocal microscope with a 25× water immersion objective (NA 0.95) and a hybrid photomultiplier tube as a detector (HYD-SMD, Leica). Cells labeled with CellTracker™ Green were excited at 492 nm, and PIC fibers grafted with Atto647N were excited at 646 nm with a nonsequential and bidirectional mode. The image stack was recorded with a distance of the confocal z-sections of 0.57 µm. With typically 175 z-slices and 512 × 512 pixels in the X–Y plane, the voxel dimension was of 0.57 µm in the x, y, and z directions. A series of time-lapse image stacks were recorded after adding cytoD (5 μM), with an interval time of 20 min between each z-stack. For collagen gel samples, fibers were imaged using 2-photon excitation at 808 nm with detection between 450 and 650 nm (550/200-nm BrightLine^®^ single-band band-pass filter, Semrock). The other image acquisition settings were the same as for the PIC samples. A stage incubator was used to keep the cells at 37 °C and 5% CO_2_ during image acquisition.

#### Displacement calculation.

The MATLAB toolbox TFMLAB was used to process the microscopy data and calculate matrix displacements around the hASCs (29). This software is freely available at https://gitlab.kuleuven.be/MAtrix/Jorge/tfmlab_public. Briefly, the workflow of TFMLAB consists of three main steps: image processing, cell segmentation, and displacement measurement.

##### Image processing.

First, raw image data were filtered by penalized least squares–based denoising and enhanced with a contrast stretching operation. Second, stage drifts were corrected for by applying rigid image registration with respect to the relaxed state. The shift was calculated by means of a phase correlation operation on the fibril images (i.e., acquired by means of fluorescence imaging for the PIC hydrogels and by means of SHG for the collagen gels). The calculated shift was then corrected for on both the fibril images and the cell images.

##### Cell segmentation.

Cell bodies were segmented by applying Otsu thresholding and by removing small binary objects (*SI Appendix*, Fig. S11 *A* and *B*).

##### Displacement measurement and 3D visualization.

TFMLAB uses the FFD-based image registration algorithm to register the stressed fibril images to the relaxed images. We used the normalized correlation coefficient as the similarity metric and a stochastic gradient descent method with adaptive estimation of the step size as the optimizer (46). As a result, a 3D displacement field vector was obtained at every voxel of the image. The software ParaView was used to create 3D renders of the cells and the displacement field using the .vtk files provided by TFMLAB.

#### Displacement field analysis.

To obtain the displacement decay away from the cell surface, we averaged the values of the displacement field magnitude at increasing distance from the cell surface with a step of 5 µm until the entire field of view was covered (*SI Appendix*, Fig. S12). Thus, an average displacement field value was obtained for each spatial bin, [0 to 5] µm, [5 to 10] µm …, and plotted in graphs shown (Figs. 2*F*, 4*F*, and 5*I*). Since the final imaging volume was approximately 230 × 230 × 100 µm^3^, the propagation of the displacements was only plotted up to 150 µm.

### Cell Morphology Analysis.

#### Automated 3D cell protrusion segmentation and cell morphology quantification.

Morphology metrics were extracted from the cell binary mask provided by TFMLAB (*SI Appendix*, Fig. S11*B*). To automatically segment cell protrusions in 3D, an in-house algorithm was developed. First, the largest sphere that fits within the cell binary mask was calculated using MATLAB functions *bwdist* and *pdist2* (*SI Appendix*, Fig. S11*C*). Letting R be the radius of the sphere, we selected the voxels from the cell binary mask located at a Euclidean distance larger than 2R. Connected components of this selection were taken as individual protrusions (*SI Appendix*, Fig. S11*D*). Protrusion length was calculated by measuring the principal axis length using MATLAB function *regionprops3.* This function was also used to compute the volume and the solidity of the cells. In this work, we refer to the solidity of the cells as “sphericity” and expressed it as a percentage.

#### Automated cell segmentation of clumped cells.

Multiple cells were present in low-magnification images. In some cases, cells generated long protrusions and were in contact with neighboring cells. First, cell and nuclei images (*SI Appendix*, Fig. S13 *B* and *C*) were filtered, enhanced, and binarized as described above (*SI Appendix*, Fig. S13*D*). To accurately split the clumped cells, the watershed algorithm from the Image Processing Toolbox dip image was applied using the nuclei masks as seeds. This provided the borders between cells in contact. After splitting the cells based on these borders, we obtained an individual mask for each cell (*SI Appendix*, Fig. S13*E*). Finally, we quantified the number of protrusions, sphericity, and volume individually as described above.

### Matrix Remodeling Analysis.

#### Image acquisition and processing.

The images were acquired using the same settings as for TFM experiment. Cells were labeled with CellTracker™ Green, and PIC fibers were grafted with Atto647N. To quantify the degree of cell-induced fiber remodeling over time, the images were acquired at different time points (0, 2, 7, 15, and 24 h) after encapsulation (*SI Appendix*, Fig. S14). To compare the matrix remodeling between stress and relaxed states, a series of time-lapse image stacks were recorded after adding cytoD (5 μM) with an interval time of 20 min (*SI Appendix*, Fig. S15). Furthermore, the raw image data were filtered by 3D Gaussian filtering.

#### Cell segmentation.

The cell was segmented by applying Otsu thresholding and by removing small binary objects.

#### Polymer segmentation.

The polymer was segmented by applying an intensity threshold based on the 99th percentile intensity of the pixels on 10% outside frame of the image. Small objects were then removed, and small holes filled.

#### Calculations.

The volume was calculated by summing all pixels inside the polymer segmented mask multiplied by the voxel size and subtracting the cell volume from it. The distance between the cell and polymer was calculated as follows: for each point on the cell mask surface, the closest point on the polymer mask surface (outer shell) was determined using Euclidean distances. We then looked at the distribution of those distances to have an estimate on how far the densified region can extend from the cell.

#### 3D modeling.

The 3D models of cell and polymer were made by plotting the isosurface for i = 0.5 which created a surface at the surface of the segmented mask.

#### Code source.

All the codes related to the matrix remodeling analysis can be found at https://github.com/BorisLouis/fiberRemodelling

### Statistical Analysis.

Statistical analysis was performed using one-way ANOVA Tukey’s test. The number of replicates for each dataset is indicated in the figure captions.

## Supplementary Material

Appendix 01 (PDF)

Movie S1. Bright field time lapse imaging showing the spreading and migration of hASCs in PIC_Short_^RGD+^, 1 mg/mL. Time is indicated in hours:minutes in the top left corner. Scale bar = 200 μm.

Movie S2. Time lapse imaging of a hASC cell in PIC_Short_^RGD+^ (1 mg/mL), after the addition of cytoD. The images were acquired every 20 min. The PIC polymers were labelled with Atto647N and are shown in green.

## Data Availability

Matlab toolbox TFMLAB has been deposited in Gitlab at https://gitlab.kuleuven.be/MAtrix/Jorge/tfmlab_public (47). The code related to the matrix remodeling analysis data has been deposited in Github at https://github.com/BorisLouis/fiberRemodelling (48). All study data are included in the article and/or *SI Appendix*.

## Footnotes

^*^Note that 150 µm limit was imposed by the size of the region imaged.
