# Supplementary material for: Synthetic fibrous hydrogels as a platform to decipher cell–matrix mechanical interactions
Source: Proc Natl Acad Sci U S A. 2023 Apr 3;120(15):e2216934120. doi: 10.1073/pnas.2216934120 (PMC10104511; doi:10.1073/pnas.2216934120)
Supplement: Supplementary file 1 — Appendix 01 (PDF) [file pnas.2216934120.sapp.pdf]

PNAS

Supporting Information for

Synthetic fibrous hydrogels as a platform to decipher cell-matrix mechanical interactions

Hongbo Yuan[a,b,1,2], Kaizheng Liu[b,c,d,1], Mar Cóndor[e], Jorge Barrasa-Fano[e], Boris Louis[b,f], Johannes Vandaele[b], Paula de Almeida[c], Quinten Coucke[b], Wen Chen[c], Egbert Oosterwijk[g], Chengfen Xing[a], Hans Van Oosterwyck[e,h], Paul H. J. Kouwer[c,2] and Susana Rocha[b,2]

[a] Key Laboratory of Molecular Biophysics of Hebei Province, Institute of Biophysics, School of Health Sciences and Biomedical Engineering, Hebei University of Technology, Tianjin 300401, China
[b] Molecular Imaging and Photonics, Chemistry Department, KU Leuven, Leuven 3000, Belgium
[c] Institute for Molecules and Materials, Radboud University, 6525 AJ Nijmegen, The Netherlands
[d] Research Center for Human Tissue and Organs Degeneration, Institute of Biomedicine and Biotechnology, Shenzhen Institute of Advanced Technology, Chinese Academy of Sciences, Shenzhen, 518055, China
[e] Department of Mechanical Engineering, Biomechanics section, KU Leuven, Leuven 3000, Belgium
[f] Division of Chemical Physics and NanoLund, Department of Chemistry, Lund University, 221 00 Lund, Sweden
[g] Radboud Institute for Molecular Life Sciences, Department of Urology, Radboud University Medical Center, 6500 HB Nijmegen, The Netherlands
[h] Prometheus Division of Skeletal Tissue Engineering, KU Leuven, Leuven 3000, Belgium
[1] H.Y. and K.L. contributed equally to this work.
[2] To whom correspondence may be addressed. Email: hongbo_yuan@hebut.edu.cn, p.kouwer@science.ru.nl, or susana.rocha@kuleuven.be.

This PDF file includes:

Figures S1 to S15
Legends for Movies S1 to S2

Other supporting materials for this manuscript include the following:

Movies S1 to S2

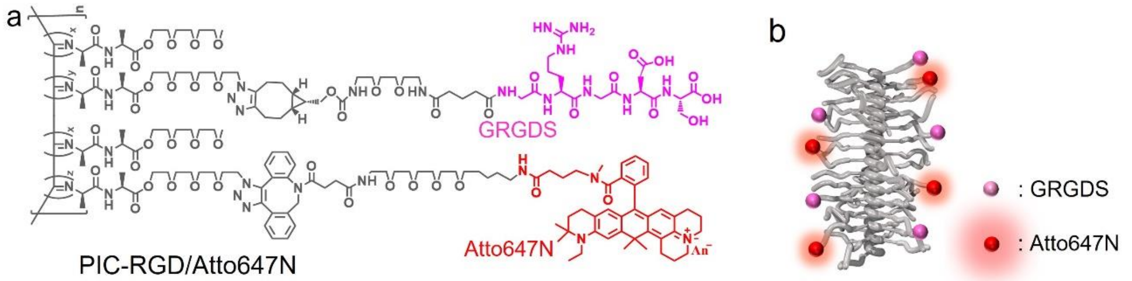

Fig. S1. (a) Chemical structure of PIC polymers decorated with GRGDS and/or Atto 647N. (b) Schematic illustration of the helical structure of PIC molecules.

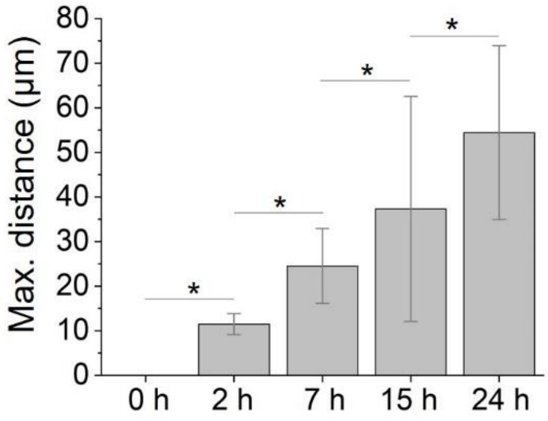

Fig. S2. Maximum distance of the detected edge of fiber remodeling to cell surface at different time points of culture (N = 5, * indicates p<0.05, by One-way ANOVA Tukey's test).

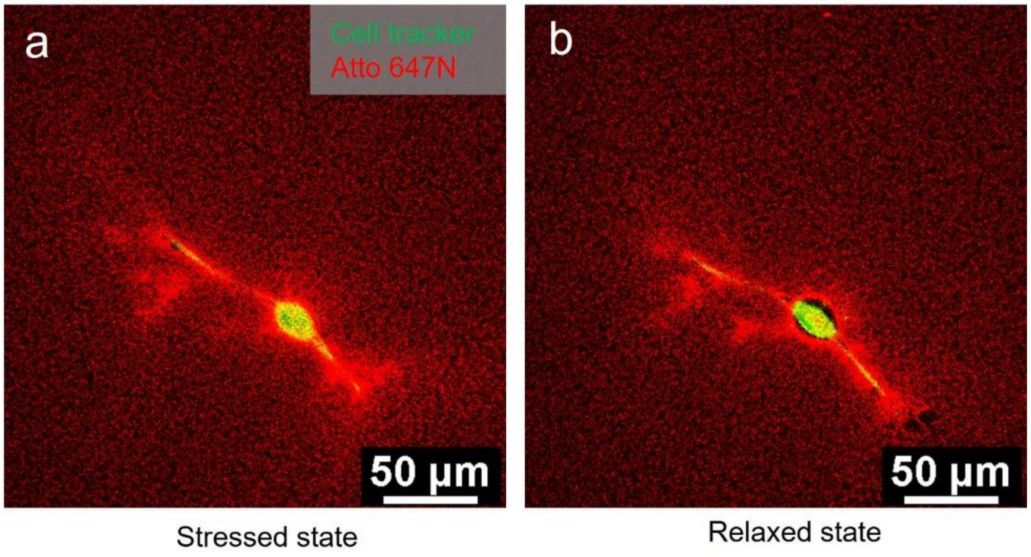

Fig S3. Schematic illustration of bead-free displacement microscopy approach based on PIC matrices. Representative fluorescence images of a cell (cell tracker in green) and surrounding PIC fibers (red) at the stress state (a), and the relaxed state (1h after adding cytochalasin D) (b).

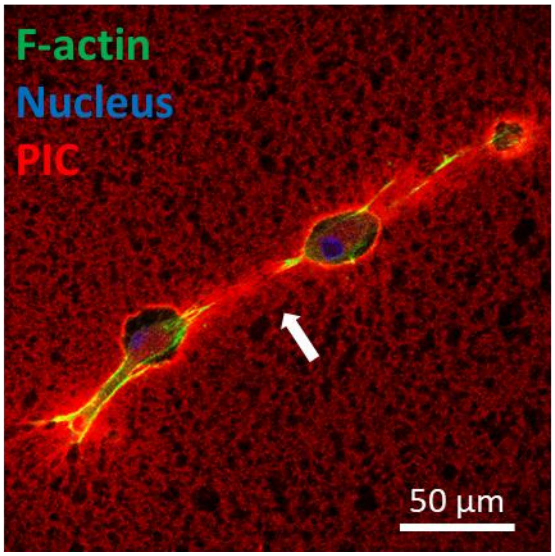

Fig. S4. Two adjacent hASCs in PIC $_{Short}^{RGD+}$ network, imaged 7 hours after encapsulation, showing fiber remodeling in the intercellular space. The remodeled fibers (white arrow) formed a track, enabling mechanical interaction between the cells. Scale bar = 50 µm.

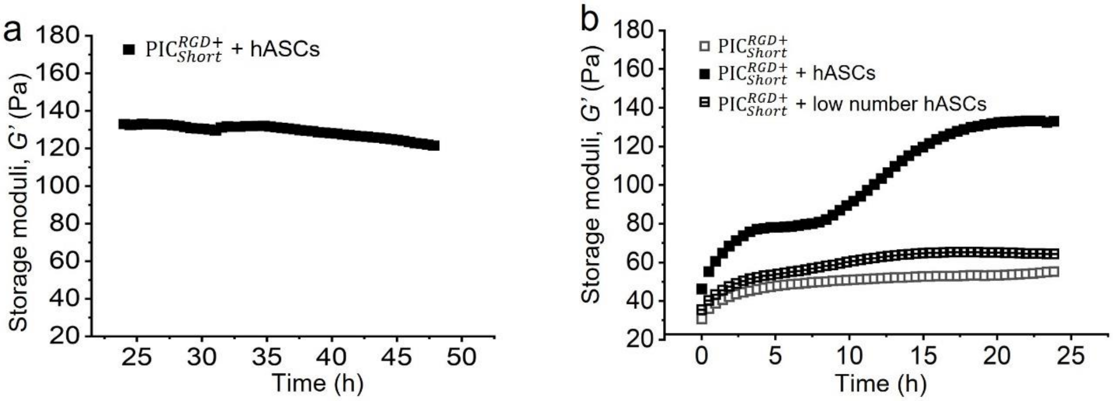

Fig. S5. Impacts of variations of other parameters on gel stiffening. (a) The stiffness of the PIC $_{Short}^{RGD+}$ hASCs-gel construct remained constant during 24-48 hours after encapsulation. (b) Influence of the cell density on matrix stiffening in PIC $_{Short}^{RGD+}$. A low cell density (250,000 cells mL^{-1}) was used. Note that in all gels, the polymer concentration was 1 mg mL^{-1}, and the cell concentration was 10^6 cells mL^{-1} unless otherwise specified.

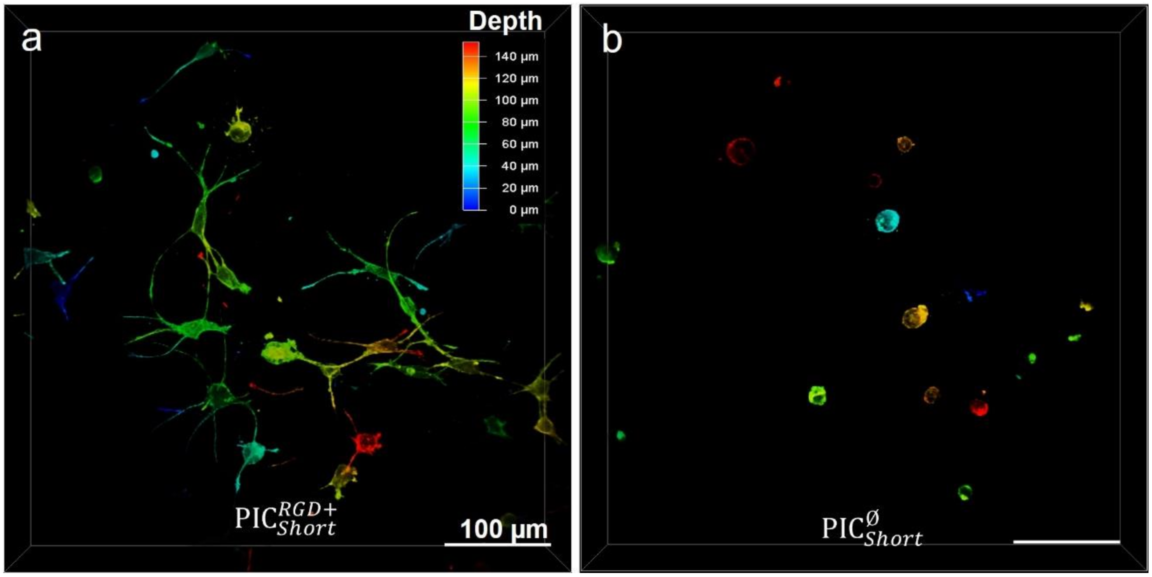

Fig. S6. Color-coded (depth) 3D confocal fluorescence images showing cell morphology in PIC gels with (a) and without (b) RGD. Cells were labeled with phalloidin.

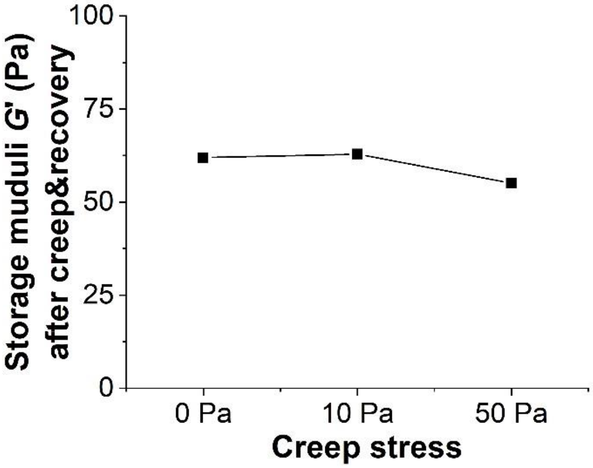

Fig. S7. The shear storage moduli of the PIC gels ($PIC^{\varnothing}_{Short}$) after a series of macroscopic creep and recovery tests with different applied stresses. The storage modulus G' was determined by applying an oscillating deformation of amplitude γ = 2%, and f = 1 Hz. The polymer concentration was 1 mg mL^{-1}.

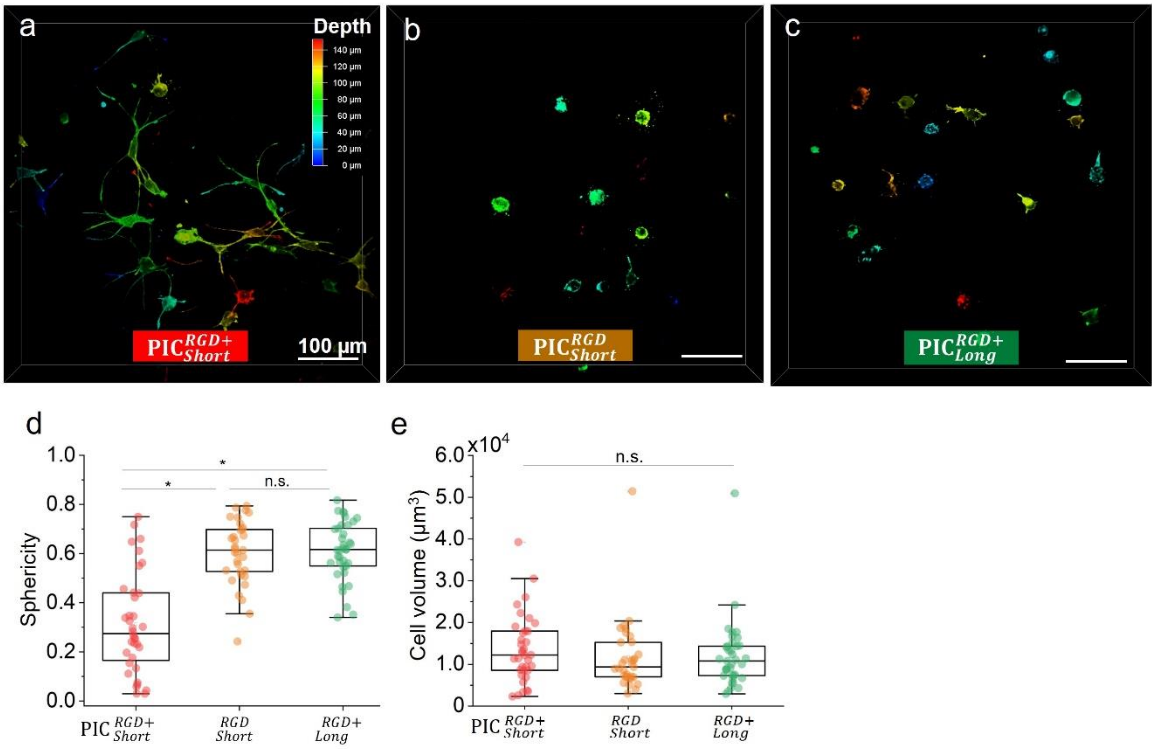

Fig. S8. Color-coded (depth) 3D confocal fluorescence images showing cell morphology in PIC gels with different conditions 24 h after encapsulation, (a) PIC $^{RGD+}_{Short}$, (b) PIC $^{RGD}_{Short}$, and (c) PIC $^{RGD+}_{Long}$. Cells were labeled with phalloidin. Analysis of cellular sphericity (d), and volume (e) in these three gels. Although cells show lower sphericity and higher number of protrusions in PIC $^{RGD+}_{Short}$ gels, protrusions are very thin and only take up a low percentage of the whole cell's volume. Hence, a comparison based on cell volume does not reveal statistically significant differences.

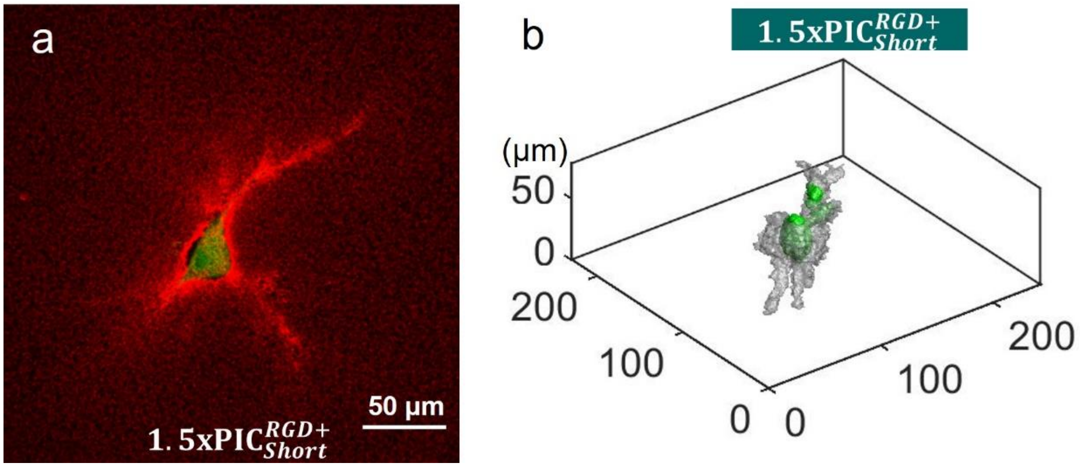

Fig. S9. Investigation of the effect of linear stiffness on matrix remodeling. (a) Fluorescence images of cell induced remodeling 24 h after encapsulation in 1.5xPIC $^{RGD+}_{Short}$ matrices (with a higher concentration at 1.5 mg/mL). Red: PIC fibers. Green: cells. (b) Representative 3D rendering of the remodeled matrix (grey) and the cell body (green).

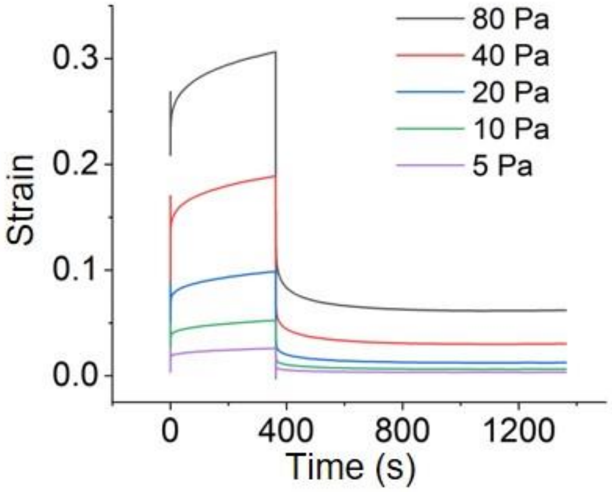

Fig. S10. Creep and recovery tests characterize plasticity of PIC gels ($PIC^{\varnothing}_{Short}$). Initially, PIC gels exhibit instantaneous elastic response to applied stress, and strain of materials gradually increases over time. At the end of the creep test, PIC gels exhibit a maximum strain. After release of the creep test, the samples undergo elastic and viscoelastic recovery, but leave an irreversible strain, which is indicative of plastic deformation.

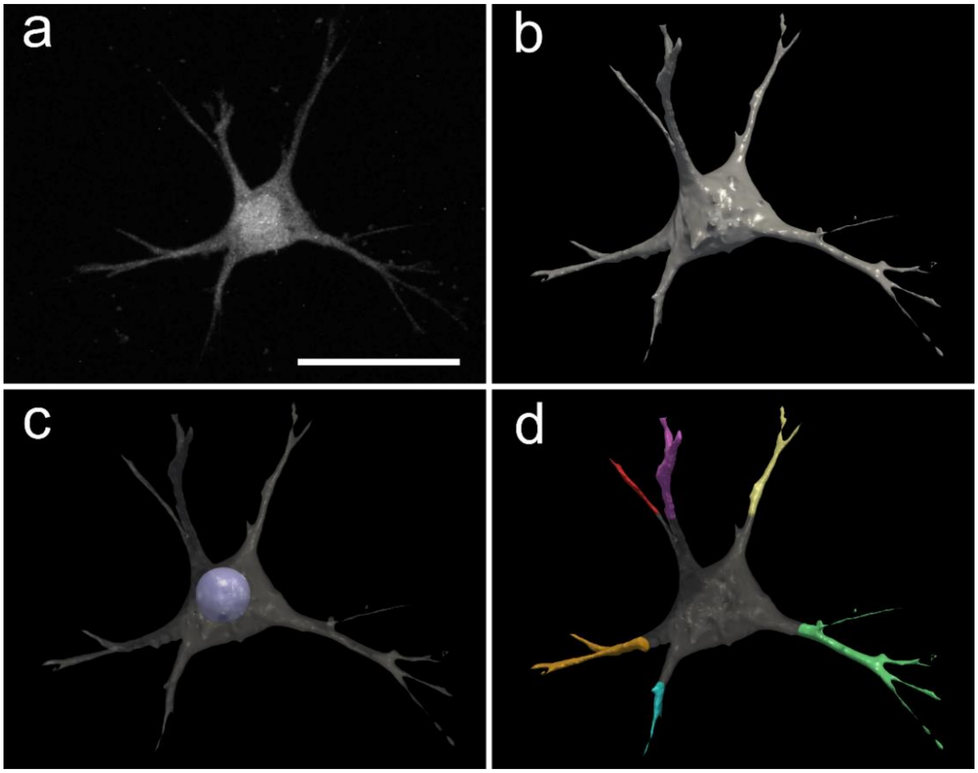

Fig. S11. Illustrative example of the automated 3D cell protrusion segmentation. (a) Maximum intensity projection of a hASC in a PIC hydrogel acquired by means of confocal microscopy. Scale bar = 45 µm. (b) 3D binary cell mask. (c) Maximum sphere (purple) within the cell mask. (d) Segmentation of the cell protrusions. Each individual protrusion is labelled in a different color. b,c and d were rendered in Paraview.

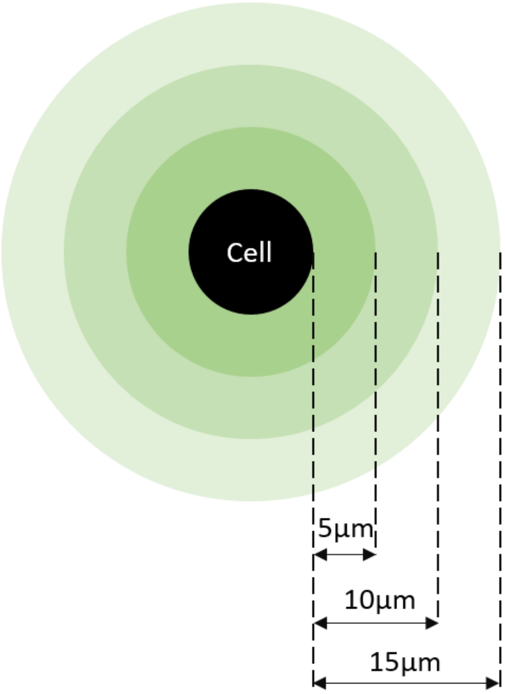

Fig S12: Schematic of the volume regions (green) away from the cell (black) that were analyzed to create the plots shown in Figs. 2f, 4f and 5i. For all the points in a given region (colored in a shade of green), the displacement field magnitude values are averaged. Please note that this is a 2D simplified representation. The analysis was done in 3D and cell morphologies were non-spherical.

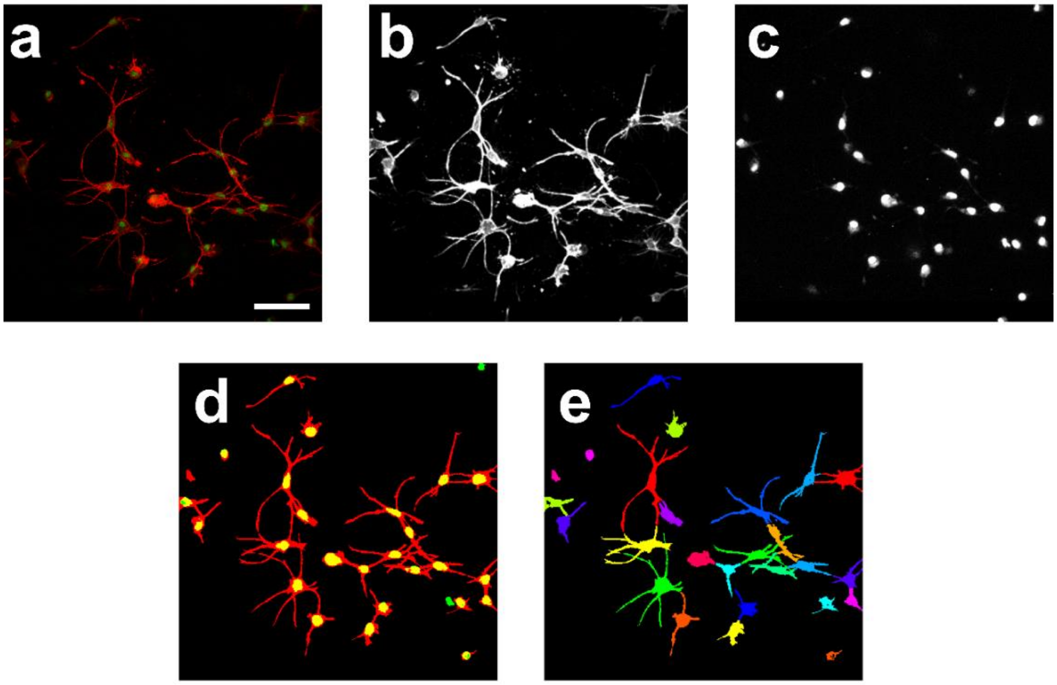

Fig. S13. Illustrative example of the automated 3D cell segmentation of clumped cells. While the images shown in this figure are maximum intensity projections, the algorithm segments 3D cell volumes. (a) Raw data contain a cell channel (red) and a nuclei channel (green). (b) Filtered and enhanced cell channel. (c) Filtered and enhanced nuclei channel. (d) Binary masks of the cells (red) and the nuclei (green). Overlap is displayed in yellow. (e) Cell splitting after applying the watershed algorithm. Individual cells are labelled in different colors. Scale bar: 100 µm.

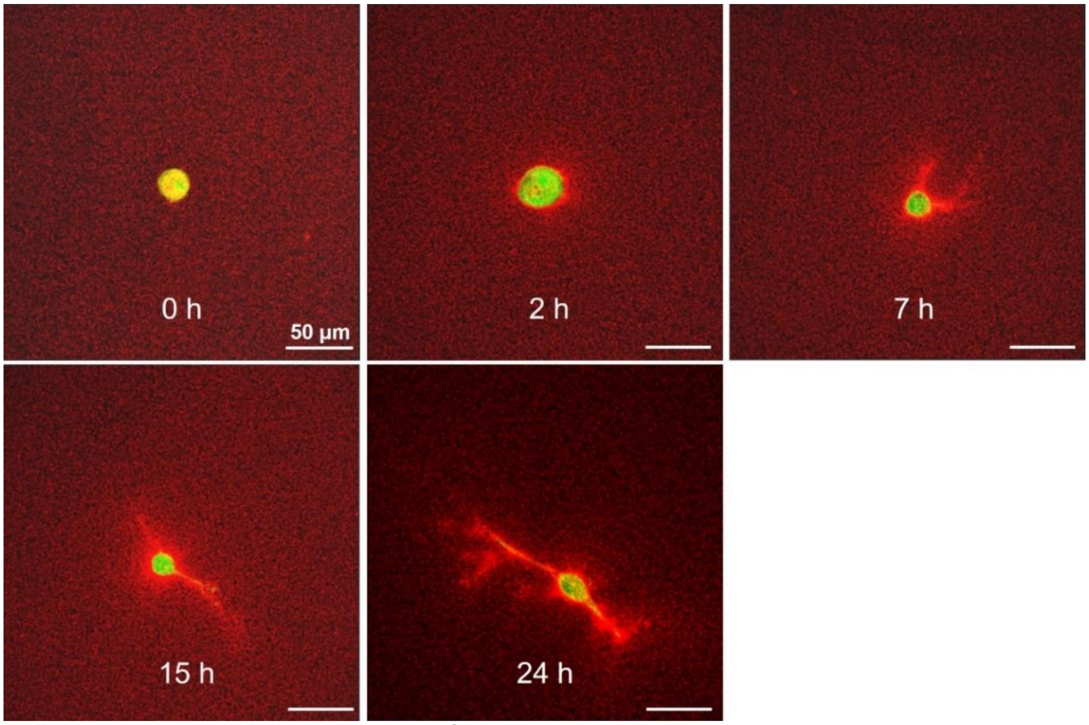

Fig. S14. Fluorescence images of PIC $^{RGD+}_{Short}$ (1.0 mg/mL) with a representative encapsulated cell at different time points from 0 to 24 hours. Red: PIC fibers. Green: cells.

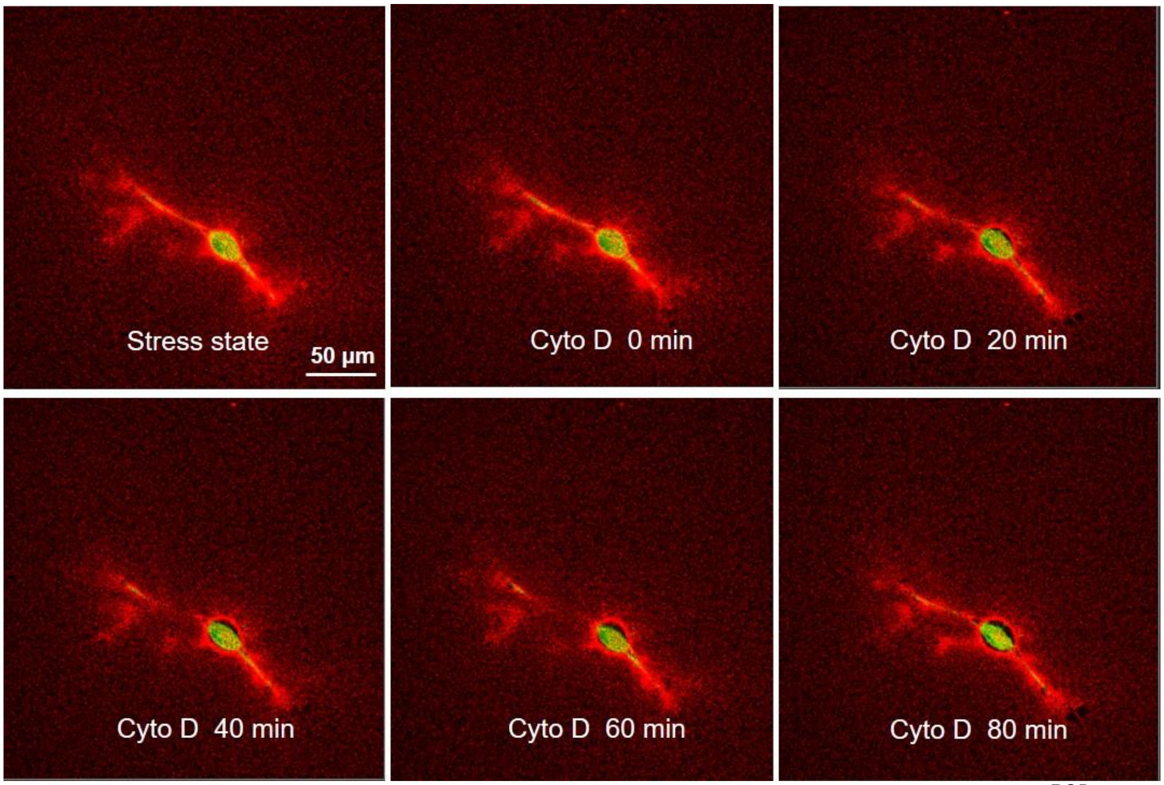

Fig. S15. Representative time-lapse images show the changes of a fiber network (PIC $^{RGD+}_{Short}$, 1.0 mg/mL) and an encapsulated cell after adding Cyto D (5 µM). Red: PIC fibers. Green: cells.

Movie S1 (separate file). Bright field time lapse imaging showing the spreading and migration of hASCs in PIC^{RGD+}_{Short}, 1 mg/mL. Time is indicated in hours:minutes in the top left corner. Scale bar = 200 µm.

Movie S2 (separate file). Time lapse imaging of a hASC cell in PIC^{RGD+}_{Short} (1 mg/mL), after the addition of cytoD. The images were acquired every 20 min. The PIC polymers were labelled with Atto647N and are shown in green.
