# Supplementary figures and images for: Synthetic fibrous hydrogels as a platform to decipher cell–matrix mechanical interactions
Source: Proc Natl Acad Sci U S A. 2023 Apr 3;120(15):e2216934120. doi: 10.1073/pnas.2216934120 (PMC10104511; doi:10.1073/pnas.2216934120)

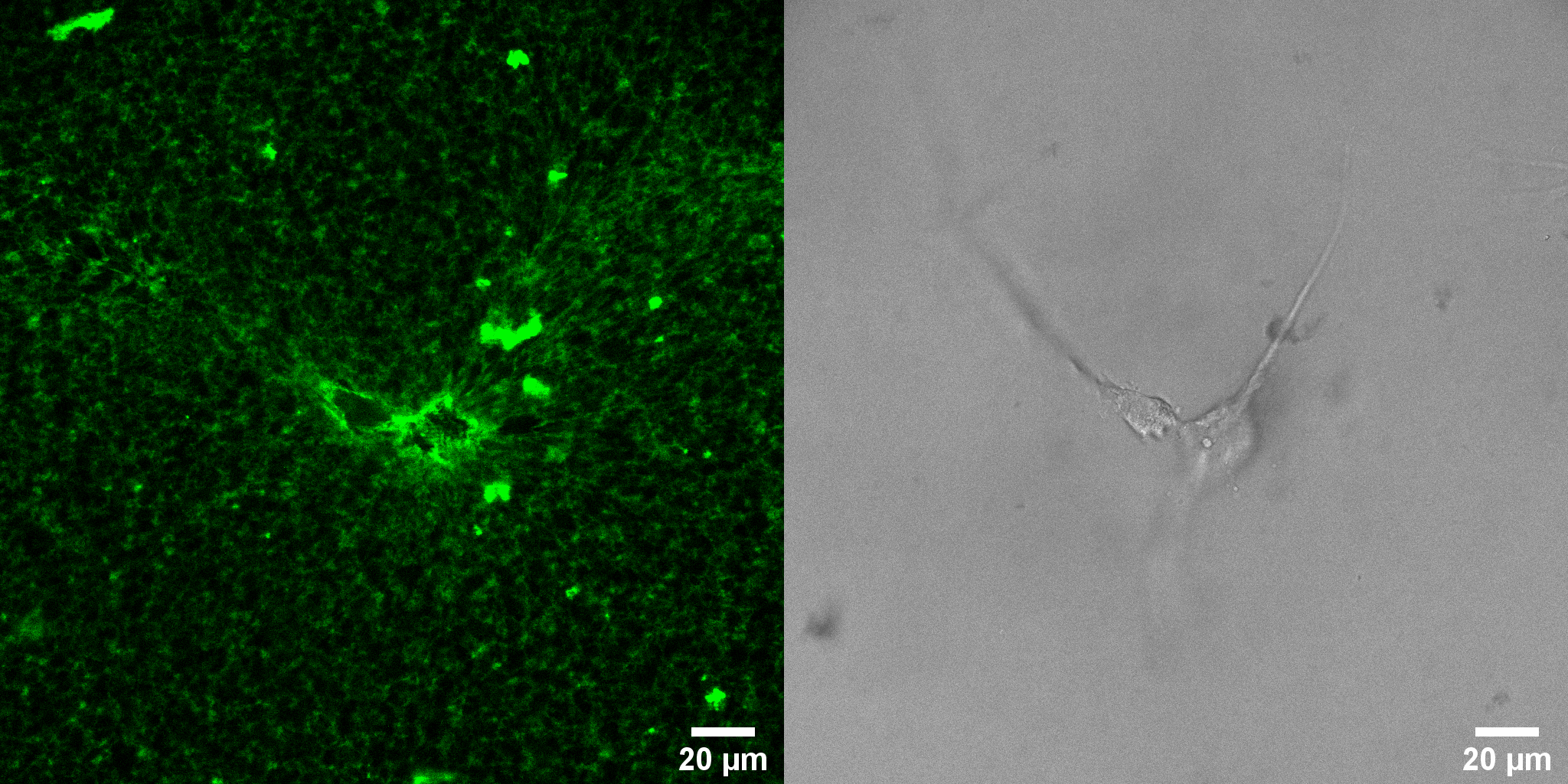

Supplement: Movie S2. — Time lapse imaging of a hASC cell in PICShortRGD+ (1 mg/mL), after the addition of cytoD. The images were acquired every 20 min. The PIC polymers were labelled with Atto647N and are shown in green. [file pnas.2216934120.sm02.gif]
